# Multimodal neuroimaging in Long-COVID and its correlates with cognition 1.8 years after SARS-CoV-2 infection: a cross-sectional study of the *Aliança ProHEpiC-19 Cognitiu*

**DOI:** 10.3389/fneur.2024.1426881

**Published:** 2024-09-13

**Authors:** Rosalia Dacosta-Aguayo, Pere Torán-Monserrat, Meritxell Carmona-Cervelló, Brenda Biaani León-Gómez, Maria Mataró, Josep Puig, Gemma Monté-Rubio, Victor M. López-Lifante, Josep Maria Manresa-Domínguez, Valeria Zamora-Putin, Pilar Montero-Alia, Carla Chacón, Jofre Bielsa-Pascual, Eduard Moreno-Gabriel, Rosa García-Sierra, M. Carmen Rodríguez-Pérez, Anna Costa-Garrido, Julia G. Prado, Eva Martínez-Cáceres, Lourdes Mateu, Marta Massanella, Concepción Violán, Noemí Lamonja-Vicente

**Affiliations:** ^1^Unitat de Suport a la Recerca Metropolitana Nord, Institut Universitari d'Investigació en Atenció Primària Jordi Gol (IDIAP Jordi Gol), Mataró, Spain; ^2^Germans Trias i Pujol Research Institute (IGTP), Badalona, Spain; ^3^Department of Medicine, Faculty of Medicine, Universitat de Girona, Girona, Spain; ^4^Multidisciplinary Research Group in Health and Society (GREMSAS) (2021-SGR-0148), Institut Universitari d'Investigació en Atenció Primària Jordi Gol (IDIAPJGol), Barcelona, Spain; ^5^Department of Clinical Psychology and Psychobiology, University of Barcelona, Barcelona, Spain; ^6^Institut de Neurociències, University of Barcelona, Barcelona, Spain; ^7^Institut de Recerca Sant Joan de Déu, Esplugues de Llobregat, Spain; ^8^Radiology Department CDI, Hospital Clinic of Barcelona, Barcelona, Spain; ^9^IDIBAPS (Instituto de Investigaciones Biomédicas August Pi i Sunyer), Barcelona, Spain; ^10^Comparative Medicine and Bioimaging Center (CMCiB), Germans Trias i Pujol Research Institute, Badalona, Spain; ^11^Palau-Solità Healthcare Centre, Palau-Solità Plegamans Institut Català de la Salut, Barcelona, Spain; ^12^Department of Medicine, Universitat Autònoma de Barcelona, Bellaterra, Spain; ^13^Grup de REcerca en Impacte de les Malalties Cròniques i les seves Trajectòries (GRIMTra), Institut Universitari d'Investigació en Atenció Primària Jordi Gol (IDIAPJGol), Barcelona, Spain; ^14^Department of Social Psychology, Universitat Autònoma de Barcelona, Bellaterra, Spain; ^15^Nursing Department, Faculty of Medicine, Universitat Autònoma de Barcelona, Barcelona, Spain; ^16^IrsiCaixa-AIDS Research, Badalona, Spain; ^17^Centro de Investigación Biomédica en Red de Enfermedades Infecciosas (CIBERINFEC), Instituto de Salud Carlos III (ISCIII), Madrid, Spain; ^18^Immunology Department, FOCIS Center of Excellence-Universitat Autònoma de Barcelona, Cerdanyola del Vallès, Spain; ^19^Immunology Division, Laboratori Clinic Metropolitana Nord (LCMN), Hospital Universitari Germans Trias i Pujol, Badalona, Spain; ^20^Infectious Diseases Department, Fundació Lluita contra les Infeccions (FLI), Germans Trias i Pujol Hospital, Badalona, Spain; ^21^Red Española de investigación en Covid Persistente, Madrid, Spain; ^22^Red de Investigación en Cronicidad, Atención Primaria y Prevención y Promoción de la Salud (RICAPPS), Instituto de Salud Carlos III (ISCIII), Madrid, Spain

**Keywords:** diffusion tensor imaging, resting state, Long-COVID, cognition, multimodal neuroimaging, connectivity

## Abstract

**Introduction:**

There is a growing interest in the effect of Long-COVID (LC) on cognition, and neuroimaging allows us to gain insight into the structural and functional changes underlying cognitive impairment in LC. We used multimodal neuroimaging data in combination with neuropsychological evaluations to study cognitive complaints in a cohort of LC patients with mild to moderate severity symptoms.

**Methods:**

We conducted a 3T brain magnetic resonance imaging (MRI) study with diffusion tensor imaging (DTI) and functional MRI (fMRI) sequences on 53 LC patients 1.8 years after acute COVID-19 onset. We administered neuropsychological tests to evaluate cognitive domains and examined correlations with Tract-Based Spatial Statistics (TBSS) and resting state.

**Results:**

We included 53 participants with LC (mean age, 48.23 years; 88.7% females). According to the Frascati criteria, more than half of the participants had deficits in the executive (59%) and attentional (55%) domains, while 40% had impairments in the memory domain. Only one participant (1.89%) showed problems in the visuospatial and visuoconstructive domain. We observed that increased radial diffusivity in different white matter tracts was negatively correlated with the memory domain. Our results showed that higher resting state activity in the fronto-parietal network was associated with lower memory performance. Moreover, we detected increased functional connectivity among the bilateral hippocampus, the right hippocampus and the left amygdala, and the right hippocampus and the left middle temporal gyrus. These connectivity patterns were inversely related to memory and did not survive false discovery rate (FDR) correction.

**Discussion:**

People with LC exhibit cognitive impairments linked to long-lasting changes in brain structure and function, which justify the cognitive alterations detected.

## 1 Introduction

Post-acute sequelae of SARS-CoV-2, known as Long-COVID (LC), is a post-viral syndrome affecting more than 30% of COVID-19 survivors (1), even those asymptomatic during the acute infection (2). Patients suffering from this syndrome develop a variety of long-lasting, debilitating symptoms, including fatigue, cognitive impairment, and depression (3), among other neuropsychiatric and physical problems. These symptoms can last for at least 3 months until more than 2 years after the infection (4) and may interfere with the patient’s quality of life and daily functioning (5).

The cognitive consequences of SARS-CoV-2 infection have been widely reported, but the brain mechanisms underlying these impairments are not well understood. A comprehensive neuropsychological evaluation of recovered COVID-19 patients who have subjective cognitive complaints could shed light on the cognitive domains affected by the infection (6–11). Attention, memory, and executive functions are the most affected cognitive domains in these patients.

Recently, several studies have explored the relationship between brain changes and cognitive impairment in patients with LC (8, 12–17). However, the long-term effects of these brain changes on cognitive function remain largely unknown, and this information could provide insights into the underlying mechanisms and outcomes of LC.

Research on the long-term effects (minimum of 3 months) of COVID-19 revealed changes in the brain’s white matter (WM) structure, including the presence of hyperintensities and alterations in microstructural integrity (18–20), although not all the studies point in the same direction One study assessed patients 1 year after SARS-CoV-2 infection and revealed reduced fractional anisotropy and volume fraction of intracellular water compared to controls (18). The same authors conducted a two-year follow-up study of the same participants and found that with some recovery, the patients with LC still showed increased radial diffusivity in some anatomical areas when compared to controls (21). In the study conducted by Liang et al. (8), 11 months after symptoms onset, they found alterations in white matter axial and mean diffusivity in different anatomical regions of the brain. In contrast, fractional anisotropy and radial diffusivity showed no significant differences between groups (8). In two studies conducted by Liang et al. and Chang et al. (13, 15) the authors found that controls and LC patients had similar cognitive performance (13, 15). In the study conducted by Liang et al. (15) LC patients showed higher fractional anisotropy (FA) and lower diffusivities in multiple white matter tracts (15). However, in the study conducted by Scardua-Silva et al. (17), the authors found higher axial diffusivity values in LC patients compared to controls (17). In the same direction, in the study conducted by Paolini et al. (16), the authors identified increased axial, radial, and mean diffusivity in LC patients with cognitive complaint in comparison with LC patients with no cognitive complaint, implicating disrupted white matter integrity in cognitive impairment during LC (16).

There are mixed findings on functional activity and connectivity. Previous research investigated the changes in brain network activity after COVID-19 infection for 3 weeks and found that the anterior piriform cortex, a region involved in olfactory processing, showed reduced functional connectivity with olfactory dysfunction (22). Díez-Cirarda et al. (23) found hypoconnectivity between left and right parahippocampal areas and between bilateral orbitofrontal and cerebellar regions compared to controls (8). In a study conducted by Churchill et al. (24) with LC patients after 11.08 ± 4.47 months since the first symptoms, they found that patients with LC had lower temporal and subcortical functional connectivity than controls (25). In another study conducted by Voruz et al. (26) they found reduced functional connectivity in COVID-19 patients compared to controls, including the hippocampal and cerebellar areas (27). Pointing to the same direction of the above-mentioned studies, the research conducted by Bungenberg et al. (12), found that the red nucleus was significantly hypoconnected across most regional graph measures. They also found decreased functional connectivity in the olfactory cortex and the medial orbital gyrus. However, they found increased connectivity in the intralaminar nuclei of the thalamus in individuals recovering from COVID-19 (12). However, studies on persistent olfactory dysfunction have reported increased connectivity within the default-mode network (DMN) (28). Another study of recovered individuals showed decreased connectivity within the temporal lobe and angular gyrus but increased connectivity within the hippocampus (29). In relation to the DMN, in a study conducted by Chang et al. (13), the LC patients exhibited greater brain activation in the right superior frontal gyrus and lesser deactivation in the default mode regions during working memory task than the control participants (13).

In the present study, we explored the long-lasting effects of COVID-19 on the brain, especially in relation to cognitive decline. To examine this issue, we used two types of brain imaging techniques by magnetic resonance imaging (MRI): diffusion tensor imaging (DTI), which measures the integrity of white matter tracts, and resting-state functional activity (rs-fMRI) and connectivity (rs-FC), which measure the synchrony of brain activity across different regions. Using a comprehensive and objective neuropsychological battery of cognitive tests, we correlated the brain images of LC patients who reported subjective complaints regarding their cognitive performance.

## 2 Materials and methods

### 2.1 Standard protocol approvals, registrations, and patient consents

The study protocol (ref.21/220-P) was approved by the Foundation University Institute for Primary Health Care Research Jordi Gol i Gurina (IDIAPJGol) ethics committee, which ensured adherence to ethical standards and guidelines. The participants gave written consent to take part in the study and to allow their data to be used for research purposes after being informed of the aims, methods, risks, and benefits of the study. All procedures followed good clinical practices and the General Data Protection Regulation 2016/679 on data protection and privacy for all individuals within the European Union.

### 2.2 Study population and design

The Aliança ProHEpiC-19 Cognitiu (APC) is a prospective, longitudinal study that explores how LC affects the brain and cognition. We recruited participants who experienced long-lasting symptoms after SARS-CoV-2 infection, with or without cognitive complaints, as well as participants who were SARS-CoV-2 infected who fully recovered, and participants who were not infected (30) from primary health centers and hospitals in Northern Area from Barcelona (Spain) between January 2022 and March 2023. Participants were recruited through hospital referrals and consecutive snowball sampling. Of the initial sample of 182 participants, 3 (1.64%) abandoned the study for various reasons. We also excluded 13 (7.14%) due to a history of neuropsychological conditions, as detailed in the exclusion criteria. Our final pool consisted of 166 participants. This paper discusses the interim findings of the first batch of 53 participants resulting from the convenience sampling of the final pool. Out of the 53 participants, 43 had mild-to-moderate COVID severity, 9 required hospitalization, but none of them required intubation or any ICU treatment (23). This cross-sectional sub-study included 53 LC patients who reported persistent cognitive complaints after SARS-CoV-2 infection, and we performed brain imaging and cognitive testing as part of the main study. We included participants with a diagnosis of LC based on WHO criteria (31), between 20 and 70 years old, and Spanish or Catalan speakers. We excluded patients with prior psychiatric, neurological, or neurodevelopmental disorders that could affect cognitive functioning, with a history of substance abuse or a life expectancy of less than 6 months, and those who were not able to undergo the MRI due to medical contraindications or claustrophobia.

### 2.3 Neuropsychological assessment and criteria for cognitive impairment

The study collected data from the participants in two sessions. In the first session, participants provided sociodemographic information detailed in the Supplementary material. Then, the cognitive domains of executive function, attention and processing speed, memory, language, and visuospatial and visuoconstructive functions were evaluated by a comprehensive neuropsychological battery administered by a trained and qualified clinical neuropsychologist with more than 5 years of experience in the assessment of neurological disorders and a psychologist carefully trained and supervised by the same clinical neuropsychologist. The cognitive tests used for each domain were as follows: for executive functions, the Digit Span Backward subtest from Pena-Casanova et al. (32, 33), a difference score (B-A) that removed the speed element from the test evaluation was calculated (34), the phonetic (letters beginning with “P,” “M,” and “R,” one minute each) and semantic verbal fluency tests (“animals” in one minute) (35, 36), and the interference score of the Color-Word Stroop Test (37); for attention and velocity, the Digit Span Forward subtest (32, 33), the Symbol Search from the WAIS-III (38), the TMT-A (32, 33), and the Symbol Digit Modality Test (WAIS-III) (38); for memory, the total learning and delayed recall from the RAVLT (39), and the delayed recall from the Rey Osterrieth Complex Figure (ROCF) (40, 41); for language, the short version of the BNT (24) and the vocabulary test from the WAIS-III (38); and for visuospatial and visuoconstructive functions, the copy accuracy of the ROCF (40, 41). Fatigue was assessed with the Modified Impact Fatigue Scale (MFIS). This scale includes three subscales: cognitive, physical, and psychosocial. In this scale, participants are asked to rate the extent of fatigue in their lives in the past 4 weeks, with “0” indicating “no problem” and “4” indicating “extreme problem.” There is a total of 21 items, 10 cognitive items, 9 physical items, and 2 psychosocial items. The maximum score is 84, 40 for the cognitive subscale, 28 for the physical subscale, and 8 for the psychosocial scale, with a score higher than 38 meaning significant fatigue (42). For each test, we used the internationally accepted adjustments and norms discussed in the corresponding papers and used the PAR Toolkit (PAR Inc. Mobile) to calculate the standard Z-scores. We used the Frascati Criteria (43) to assess cognitive impairment in participants with LC. According to these criteria, patients have cognitive impairment if they score below −1.5 standard deviation (SD) across the sample on any subtest within a cognitive domain or below −1 SD on 2 subtests of the same cognitive domain.

In the second session, LC individuals underwent a brain MRI scan acquisition within 6 months (*x̅* = 6.23; SD = 4.20) from the cognitive assessment. To protect the privacy of our participants, we used cryptographic hashtags to anonymize the project database. We also employed the same 10-digit numeric encoding system hosted by REDCap, version 12.4.22—Vanderbilt University, for the MRI study.

### 2.4 Neuroimaging

In the second session, LC individuals underwent a brain MRI scan acquisition within 6 months (*x̅* = 6.23; SD = 4.20) from the cognitive assessment. To protect the privacy of our participants, we used cryptographic hashtags to anonymize the project database. We also employed the same 10-digit numeric encoding system hosted by REDCap, version 12.4.22—Vanderbilt University, for the MRI study.

#### 2.4.1 Neuroradiological assessment of the structural magnetic resonance imaging

The assessment was carried out by the project’s neuroradiologist with extensive experience, primarily aimed at excluding the presence of brain lesions. This was achieved through the examination of T1-weighted, FLAIR, and diffusion sequences, with a specific focus on identifying regions of encephalomalacia due to trauma or previous surgical interventions, territorial vascular infarctions, lacunes, or brain tumors. No incidental findings were identified that needed specific medical attention or further secondary testing in our cohort. Five individuals had isolated and non-specific hyperintensities on FLAIR in the supratentorial subcortical white matter.

#### 2.4.2 MRI acquisition protocol

All images were acquired on a Vantage Galan 3T MRI (Canon Medical Systems Corporation, Tochigi, Japan) at the Centre for Comparative Medicine and Bioimage Image (CMCiB, Germans Trias i Pujol Research Institute [IGTP], Badalona, Spain) using a 32-channel head SPEEDER coil with foam padding and headphones to limit head movement and suppress scanner noise. See Supplementary material for the MRI protocol and acquisition and parameters.

#### 2.4.3 Processing and analysis of the diffusion-weighted images

The diffusion-weighted images were processed using FSL’s FMRIB Diffusion Toolbox (FDT).^1^ We used the two B0 images with opposite phase-encoding directions to run topup^2^ and eddy^3^ tools to estimate and correct susceptibility-and motion-induced distortions. After estimating the diffusion tensor models, we calculated the maps of fractional anisotropy (FA), radial diffusivity (RD), and axial diffusivity (AD). We entered them into Tract Based Spatial Statistics—TBSS (44) to test whether they have a significant relationship with performance in the different cognitive domains (each domain in separate models). Inference was calculated using nonparametric analysis using 10,000 permutations and Threshold-Free Cluster Enhancement (TFCE), adjusting for age, sex, and years of education. We excluded vascular risk factors and body mass index (BMI) from the model because they did not show any significant association with the cognitive outcome variables in the regression analysis (see the Supplementary material for the statistical models). The resulting *p*-values were corrected for Family-Wise Error (FWE). With a threshold of 0.05.

#### 2.4.4 Processing and analysis of the resting-state fMRI

The preprocessing of the functional data was conducted using FEAT (FMRI Expert Analysis Tool, v.6) from FSL-6.0.6 (45).^4^ Briefly, the first five volumes were removed to ensure the adaptation of the participants to the imaging environment, leaving 251 volumes for analysis. Further steps included the removal of non-brain structures using the Brain Extraction Tool, motion correction using MCFLIRT, high-pass filtering with a frequency cut-off at 100 s, spatial smoothing using a Gaussian kernel with full-width half-maximum of 5 mm, and boundary-based registration to the subject’s anatomical T1-weighted image, and linear registration to the MNI152 template. After this preprocessing, we used ICA-AROMA (46), a data-driven method, to identify and remove motion-related components. After preprocessing, we run FSL MELODIC (version 3.15) using the temporal concatenation approach to identify 25 independent components (IC). The clinical relevance of the ICs was tested using dual regression (47, 48) and nonparametric testing with 10,000 random permutations (49). The model included the cognitive performance (each domain in separate models) as a regressor of interest and was adjusted for age, sex, and years of education. After employing threshold-free cluster enhancement (TFCE), regression with a family-wise error (FWE) corrected *p* < 0.05 was considered significant. ICs with significant regression were anatomically labeled with reference to the Harvard-Oxford cortical and subcortical structural atlases^5^ and matched to the resting-state network template available online (50, 51) using spatial correlation and visual inspection and to annotate them both anatomically and functionally.

#### 2.4.5 Functional connectivity analysis

We used FreeSurfer to parcellate the cortex and subcortex based on the Desikan-Killiany atlas and aligned it with fMRI data in the subject space, which resulted in 91 regions of interest (ROI, after excluding white matter, CSF, ventricles, and cerebellum). We extracted and averaged the time series from each ROI and computed the Pearson’s correlation coefficients between the right and left hippocampus and the other 90 ROIs, resulting in 90 correlations per hippocampus. We converted the correlation coefficients to Fisher’s Z-transformed scores and tested whether they varied across the participants with memory performance while controlling for age, sex, and education. We applied FDR correction to the *p* ≤ 0.05 values to account for the multiple testing.

### 2.5 Statistical analysis

Statistical analyses on demographical data were performed with the Statistical Package for the Social Sciences (SPSS, Chicago), version 17.0 for Windows. The distributions of demographic variables were tested for normality by the Shapiro–Wilk test. Continuous variables were expressed as mean, standard deviation, or median and interquartile range. Categorical variables were defined as frequencies and percentages. Results from the cognitive tests were z-scored before the analysis, considering age and years of education for the main tests.

## 3 Results

### 3.1 Demographical and clinical characteristics

This study included 53 individuals with LC and cognitive complaints with a mean age of 48.23 years (SD = 9.2) and a mean education level of 14.04 years (SD = 2.6). Most participants were female (88.7%) and had a mild–moderate clinical spectrum of COVID-19 (81.1%). The sample’s most common vascular risk factors were smoking (current or former, 44.2%) and alcohol consumption (40.4%), the 34% of participants were overweight (BMI 25–29.9), 13.2% were obese (BMI 30–34.9), and 13.2% were extremely obese (BMI ≥ 35). The mean time since diagnosis of COVID-19 was 1.8 years (SD = 0.52). The 92.45% (*n* = 49) of participants were vaccinated. Diagnosis of COVID-19 was as follows: 66.04% (*n* = 35) Polymerase chain reaction (PCR); 7.55% (*n* = 4); Rapid antigen test (TAR); 9.43% (*n* = 5) serology; and 16.98% (*n* = 9) symptomatology.

The most frequent persistent symptoms were weakness and discomfort (92.5%) and fatigue (83%), followed by nonspecific insomnia (80%), muscle pain (71.9%), vertigo and dizziness (71.2%), and tingling sensations (66.7%). The most common cognitive symptoms reported were difficulty with concentration and memory (96.2%), followed by brain fog (84.9%). 92.5% of the sample scored more than 38 points on the Modified Impact Fatigue Impact Scale (MFIS) (23, 42).

### 3.2 Cognitive characteristics

Taking into consideration cognitive domains and following Frascati criteria, the most impaired cognitive domains were executive function (58.5%), attention and processing speed (54.7%), and memory (39.6%). Only one participant showed problems in the visuospatial and visuoconstructive function (1.9%). None of them had difficulties in the language domain. The most impaired cognitive tests (scores below −1.5 SD) were semantic verbal fluency (41.5%), digit span forward (33%), and phonological verbal fluency (32.1%) (23).

### 3.3 Diffusion tensor imaging

**Figure 1:**
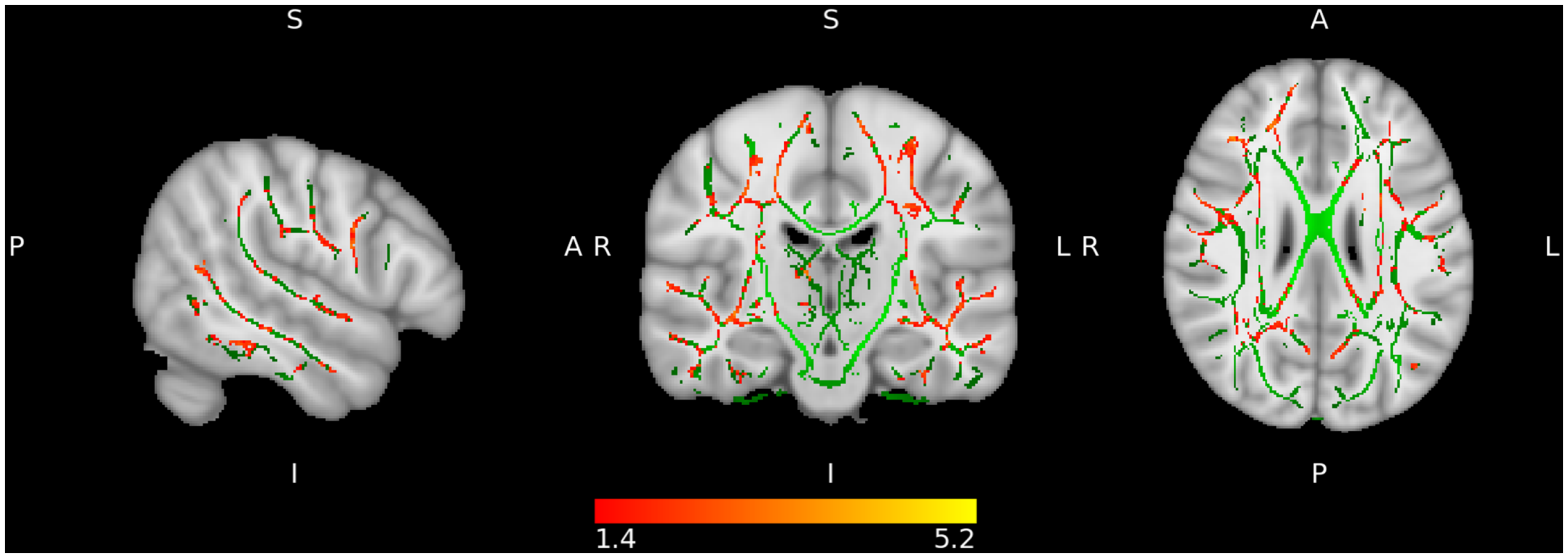
Increased radial diffusivity (RD) (red-yellow) superimposed on the tract skeleton (green).

LC participants showed a significant regression with a negative slope with t > 1.4 between radial diffusivity (RD) and memory performance in a widely distributed network of many tracts (Table 1 and Figure 1). No tract showed significant regression for fractional anisotropy (FA) or axial diffusivity (AD).

**Table 1:** Significant White Matter RD negatively correlated with memory.

Structure	Voxel	t-Max	*X*	*Y*	*Z*	*p*-value
Anterior thalamic radiation, L	1,512	4.1	−22.72	6.68	13.87	0.029
Anterior thalamic radiation, L	7	2.2	16.26	−38.17	32.74	0.029
Anterior thalamic radiation, R	5	2.06	−23.25	−13.13	2.61	0.029
Anterior thalamic radiation, R	1,163	3.74	18.25	−0.32	13.04	0.029
Corticospinal tract, L	1,216	3.69	−21.12	−23.83	41	0.029
Corticospinal tract, L	14	2.24	20.5	−7.08	7.1	0.029
Corticospinal tract, R	1,655	4.54	21.15	−23.22	39.06	0.029
Cingulum (cingulate gyrus), L	653	3.35	−12.98	−33.39	29.11	0.029
Cingulum (cingulate gyrus), R	366	3.5	15.53	−38.05	33.83	0.029
Cingulum (hippocampus), L	142	3.36	−29.09	−8.4	−30.91	0.029
Cingulum (hippocampus), L	14	2.38	15.79	−61.27	42.82	0.029
Cingulum (hippocampus), R	443	4.51	24.46	−33.31	−6.17	0.029
Forceps major, L	19	2.24	−26.84	−67.71	8.01	0.029
Forceps major, R	310	3.5	18.52	−49.14	17.6	0.029
Forceps minor, L	451	4.21	−14.56	41.03	12.69	0.029
Forceps minor, R	1,016	3.69	16.31	47.82	14.59	0.029
Inferior fronto-occipital fasciculus, L	1,333	4.06	−32.88	−9.37	−0.49	0.029
Inferior fronto-occipital fasciculus, R	1983	4.28	31.19	−21.38	5.01	0.029
Inferior longitudinal fasciculus, L	2,120	3.87	−40.86	−20.67	−12.87	0.029
Inferior longitudinal fasciculus, R	2,106	4.23	41.1	−23.87	−11.64	0.029
Superior longitudinal fasciculus, L	4,727	4.41	−40.23	−21.48	21.56	0.029
Superior longitudinal fasciculus, R	4,327	5.12	39.03	−20.91	27.66	0.029
Uncinate fasciculus, L	619	3.7	−31.15	12.25	−12.64	0.029
Uncinate fasciculus, R	513	3.5	27.75	13.29	−16.32	0.029
Superior longitudinal fasciculus, L	73	3.19	−44.44	−28.79	2.06	0.029
Superior longitudinal fasciculus, R	389	3.13	49.01	−37.38	−1.65	0.029

### 3.4 Resting-state fMRI

**Figure 2:**
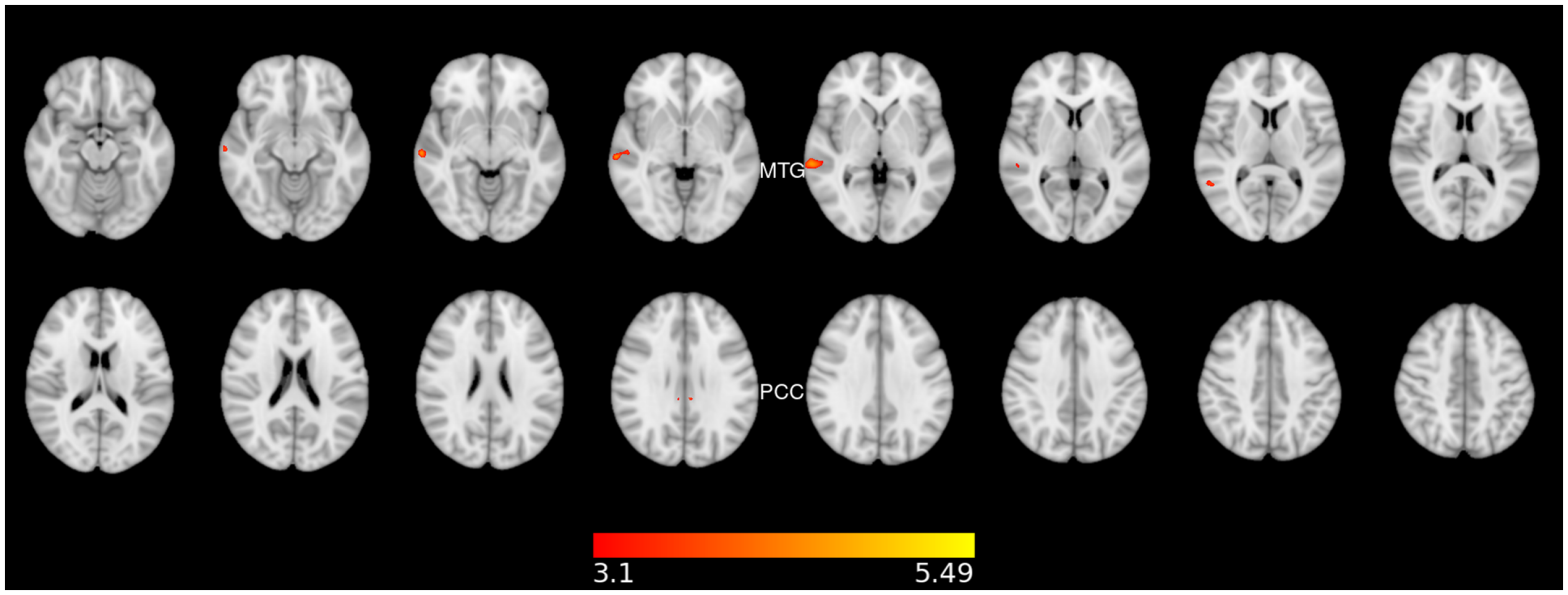
Significant regression with a negative slope between the memory performance and the resting-state fMRI activity in the right fronto-parietal network. MTG, middle temporal gyrus; PCC, posterior cingulate cortex right and left. The color bar shows the T-scores.

**Figure 3:**
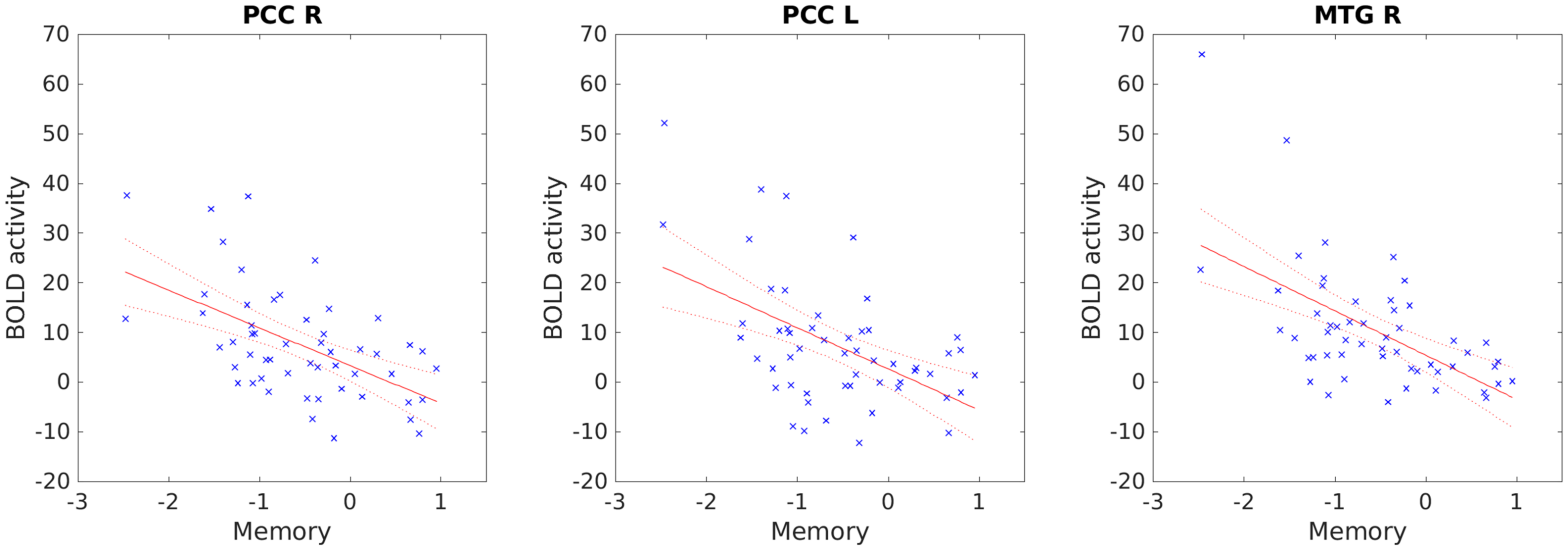
Negative regression between memory performance and resting-state activity in the fronto-parietal network. Abbreviations: act, resting-state fMRI activity; MEM, memory domain; MTG_R, middle temporal gyrus, right; PCC_R, posterior cingulate cortex, right; PCC_L, posterior cingulate cortex, left.

LC participants showed a significant regression with a negative slope with *t* > 3.1 between memory performance and the resting-state fMRI (rs-fMRI) activity in the Independent component (IC) corresponding to the right fronto-parietal network (FPN). Three clusters with local maxima in the right middle temporal gyrus (further involving the superior temporal gyrus and the supramarginal gyrus) and the right and left posterior cingulate cortices showed significant regression (Figure 2 and Table 2), meaning more impairment in the memory domain was related to greater activation in these regions (Figure 3).

**Table 2:** Anatomical regions with significant resting state activity.

Structure	Voxels mm^3^	t-Max	*X*	*Y*	*Z*	*p*-value
Sup. Temporal Gyrus, posterior division, R	179	4.9	59.27	−27.9	0.63	0.001
Mid. Temporal Gyrus, posterior, division, R	137	5.43	64.32	−25.55	−5.05	0.001
Mid. Temporal Gyrus, temporooccipital, R	70	4.34	57.66	−47.37	7.59	0.001
Supra. Gyrus, posterior division, R	11	3.84	54.15	−38.9	7.1	0.001
Cing. Gyrus, posterior division, L	17	4.43	−4.7	−33.4	28.81	0.001
Cing. Gyrus, posterior division, R	15	4.67	6.25	−33.06	27.19	0.001

Sup, Superior; Mid, Middle; Supra, Supramarginal; Cing, Cingulate.

### 3.5 Resting-state functional connectivity (rs-FC)

**Figure 4:**
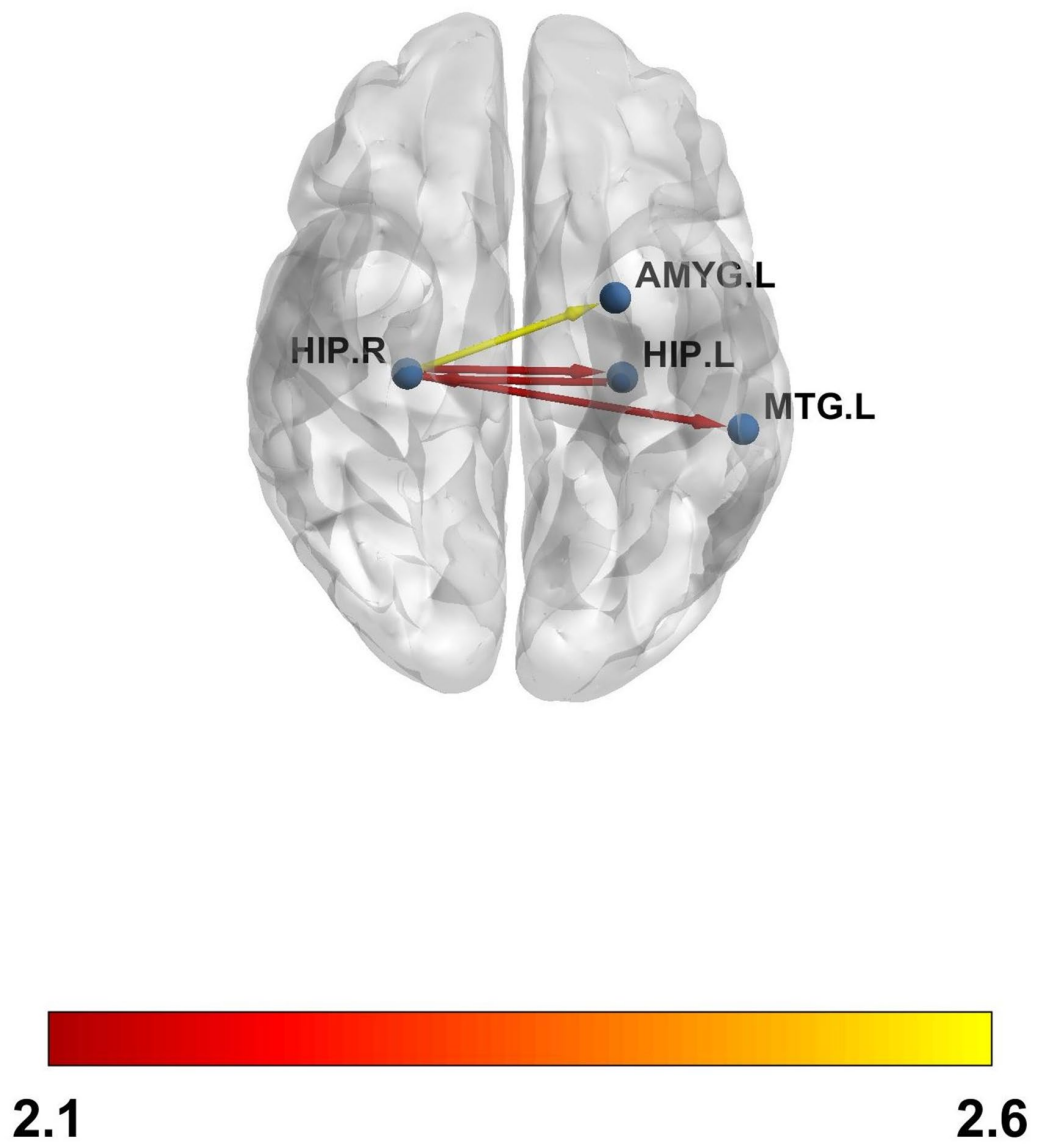
Resting-state functional connectivity (rs-FC) between the left and the right hippocampus, the right hippocampus and the left amygdala, and the left middle temporal gyrus. The color bar shows the T-values. The *p* values did not survive FDR.

**Figure 5:**
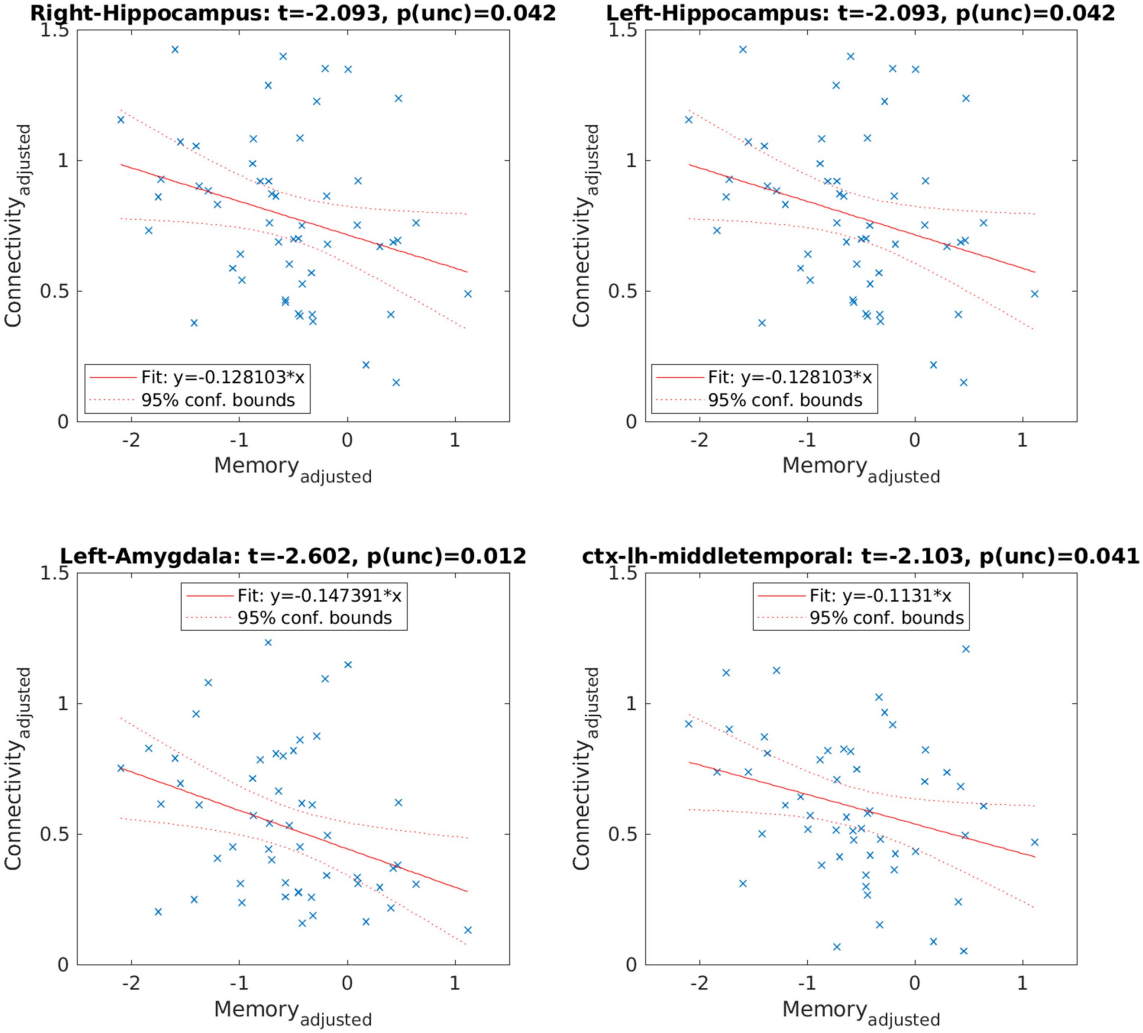
Negative regression between memory performance and the right and left hippocampal functional connectivity with the aforementioned anatomical regions.

Functional connectivity of the hippocampi with several cortical and subcortical regions showed a negative regression with memory performance (Figure 4 and Table 3), meaning higher impairment in the memory domain was related to higher functional connectivity between the corresponding hippocampus (Figure 5). No significant *p*-value overcame the FDR correction.

**Table 3:** Resting-state functional connectivity (rs-FC) correlations.

	Right hip	Left hip	Left amyg	Left MTG
Left hip	*β* = −0.13; *t* = −2.1; punc = 0.04			
Right hip			*β* = −0.15; *t* = −2.6; punc = 0.01	*β* = −0.11; *t* = −2.1; punc = 0.04

## 4 Discussion

In this study, we evaluated the brain changes in structure and function that were observed in 53 LC participants who had cognitive complaints almost 2 years after they were infected with SARS-CoV-2. Our study revealed that the participants experienced a wide range of physical and cognitive symptoms, with weakness, discomfort, and fatigue being the most prevalent. Other physical symptoms included non-specific insomnia, muscle pain, vertigo and dizziness, and tingling sensations. The cognitive symptoms reported by the participants matched the findings of previous research in this area (6–11). However, those findings did not match the results found in the study conducted by Liang et al. (15) in which the authors found that controls and LC patients had similar cognitive performance (15). Nevertheless, LC patients had higher levels of depression, anxiety, fatigue, pain interference, and perceived stress, and it did not seem to affect the cognitive results as one could expect. Emotional stress was not assessed in our study. Therefore, no interpretations can be made on this point. However, preliminary results on this point are controversial as some authors (52) have found that it is closely related to subjective cognitive complaints and could interfere with cognitive performance, while others (15) reported near-normal cognitive performance despite the presence of depression, anxiety, and perceived stress. Our analysis showed a negative correlation between memory domain performance and RD across multiple WM tracts. RD is a measure of how easily water molecules can move across the axons of nerve cells. We observed that lower scores in memory were associated with RD values in our cohort (i.e., negative correlation), suggesting reduced myelination of the axons and that memory impairment was linked to lower integrity of the white matter network. This effect was distributed symmetrically in both hemispheres. Those results are in agreement with a two-year longitudinal study conducted by Huang et al. (21) in which the authors reported that although there was a recovery over time, there were still signs of inflammation and a disrupted RD in the participants with LC compared to a group of healthy controls (21). In this direction, multiple sclerosis (MS) has been proposed as a model to study the effects of SARS-CoV-2 on brain atrophy (53). In patients with MS, RD is often increased, indicating damage to the nerve cells. This damage can affect cognitive functions, such as memory, attention, and reasoning. Previous studies have shown that increased RD is strongly related to cognitive impairment in MS (54, 55). In this line, RD was negatively correlated with a decline in memory in our LC cohort. Recent studies (15) have highlighted the impact of COVID-19 on brain structure, revealing that LC patients exhibit abnormal brain diffusivity, including changes in white matter integrity, such as patients including differences in fractional anisotropy (FA) and mean diffusivities in various white matter tracts, suggesting possible neuroinflammation or changes in myelination (15).

Regarding rs-fMRI, we found a significant independent component, the fronto-parietal network (FPN), which was negatively correlated with the cognitive memory domain. That is, greater impairment in the memory domain was related to greater activation in these regions. The regions that showed more activation were the right middle temporal gyrus and right and left posterior cingulate cortices. The right middle temporal gyrus (rMTG) is a brain region implicated in various cognitive functions, such as language processing, semantic memory, and social cognition. Recent studies have suggested that the rMTG is involved in the integration of multimodal information, such as visual, auditory, and linguistic cues, to form coherent representations of concepts and events. The rMTG may also play a role in the modulation of attention and salience, as well as in the detection of incongruence and conflict between different sources of information (56). The posterior cingulate cortex (PCC) is also a core component of the default mode network (DMN), a large-scale brain system that supports self-referential, social, and memory-related functions. The PCC has been implicated in various aspects of DMN processing, such as switching between internal and external attention, monitoring the relevance of stimuli, and integrating information across modalities and domains (26, 57). Resting-state increases in the middle temporal gyrus (right and left) and the posterior cingulate cortex have been related to subcortical vascular mild cognitive impairment (MCI) (58). According to recent studies, COVID-19 is significantly associated with different types of vascular impairment. The virus can affect blood vessels and cause inflammation, clotting, or bleeding (59–63). Although FPN hypometabolism has been proposed as a biological fingerprint of LC neurocognitive impairment (64), we propose that the unusually elevated neural activity observed in patients with LC at rest may indicate a compensatory mechanism that partially mitigates the memory impairment associated with their condition. This hypothesis is based on the assumption that increased activity may reflect enhanced encoding or retrieval processes that facilitate memory performance in the absence of external stimuli. Interestingly, in the study conducted by Chang et al. (13) the authors suggest an alternative compensatory mechanism when LC patients with neuropsychiatric symptoms heavily use alternative brain regions and networks to maintain normal performance during working memory tasks (13).

Finally, regarding the results from the rs-FC, we observed that, although no *p* ≤ 0.05 value supported FDR correction for multiple testing, we observed higher functional connectivity between the left and right hippocampus, the right hippocampus and the left amygdala, and between the right hippocampus and the right middle temporal gyrus in those with lower memory performance. That is, the worse the memory, the higher the connectivity. The results reported in the literature regarding functional connectivity in LC patients are controversial. Some authors have found hypoconnectivity between different anatomical regions (8, 25, 27), whereas others have found hyperconnectivity (28, 29). In particular, Cattarinussi et al. (54) found hyperconnectivity in the hippocampus, a region in which we also observed hyperconnectivity (29). Our results, although meaningful in the context of memory, should be interpreted with caution and validated with more samples, as they did not support the FDR correction.

One of the strengths of our study compared to previous research is that we evaluated LC and cognitive complaints in participants who had mild to moderate SARS-CoV-2 infection 1.8 years after symptom initiation and in a relatively middle-aged population. In addition, we identified brain structural and functional abnormalities related to cognitive performance. These abnormalities would not have been visible with conventional MRI within the healthcare system as we applied a comprehensive research-oriented MRI protocol. Our results present a robust and systematic methodological approach to investigate the neural and cognitive effects of LC. We used multiple neuroimaging methods and a comprehensive cognitive battery to assess LC patients, most of whom were outpatients, reflecting the typical COVID-19 population.

However, it also has some limitations that merit comment. First, our sample size may not be sufficient to generalize these results to this population type. Second, the lack of control groups prevents establishing the specificity of our findings. Therefore, future research should follow three directions: first, MRI assessment should be done for a wider population of LC individuals with cognitive complaints and compare them to control groups. Third, these studies should be repeated over time to see if these structural changes are stable, progressive, or reversible. Fourth, cognitive interventions should be evaluated in LC to see if they affect cognition and its neural correlates.

The present study used a comprehensive neuropsychological battery and a highly specialized MRI protocol to investigate brain white matter integrity and resting state functional activity and their associations with cognitive function in middle-aged participants. Our results showed that the cognitive deficits in memory were associated with a disruption in radial diffusivity, an increase in resting state activity, and functional connectivity in different anatomical regions, which may explain the cognitive complaints described by these participants.

## Data Availability

The raw data supporting the conclusions of this article, may be made available upon reasonable request to the authors of the article.

## Footnotes

^1^https://fsl.fmrib.ox.ac.uk/fsl/fslwiki/FDT

^2^https://fsl.fmrib.ox.ac.uk/fsl/docs/#/diffusion/topup/index

^3^https://fsl.fmrib.ox.ac.uk/fsl/docs/#/diffusion/eddy/index

^4^http://www.fmrib.ox.ac.uk/fsl

^5^http://fsl.fmrib.ox.ac.uk/fsl/fslwiki/Atlases

## References

[1] Tenforde MW Kim SS Lindsell CJ Rose EB Shapiro NI Clark Files D. Symptom duration and risk factors for delayed return to usual health among outpatients with COVID-19 in a multistate health care systems network — United States, march–June 2020. MMWR Morb Mortal Wkly Rep. (2020) 69:993–8. doi: 10.15585/mmwr.mm6930e1, PMID: 32730238 PMC7392393

[2] Huang C Huang L Wang Y Li X Ren L Xiaoying G. 6-month consequences of COVID-19 in patients discharged from hospital: a cohort study. Lancet. (2023) 401:e21–33. doi: 10.1016/S0140-6736(23)00810-3, PMID: 37321233 PMC10258565

[3] Renaud-Charest O Lui LMW Eskander S Ceban F Ho R Di Vincenzo JD. Onset and frequency of depression in post-COVID-19 syndrome: a systematic review. J Psychiatr Res. (2021) 144:129–37. doi: 10.1016/j.jpsychires.2021.09.054, PMID: 34619491 PMC8482840

[4] Nalbandian A Sehgal K Gupta A Madhavan MV McGroder C Stevens JS. Post-acute COVID-19 syndrome. Nat Med. (2021) 27:601–15. doi: 10.1038/s41591-021-01283-z, PMID: 33753937 PMC8893149

[5] Mateu L Tebe C Loste C Santos JR Lladós G López C. Determinants of the onset and prognosis of the post-COVID-19 condition: a 2-year prospective observational cohort study. Lancet Reg Health. (2023) 33:100724. doi: 10.1016/j.lanepe.2023.100724, PMID: 37954002 PMC10636281

[6] Ariza M Cano N Segura B Adan A Bargalló N Caldú X. COVID-19 severity is related to poor executive function in people with post-COVID conditions. J Neurol. (2023) 270:2392–408. doi: 10.1007/s00415-023-11587-4, PMID: 36939932 PMC10026205

[7] Delgado-Alonso C Valles-Salgado M Delgado-Álvarez A Yus M Gómez-Ruiz N Jorquera M. Cognitive dysfunction associated with COVID-19: a comprehensive neuropsychological study. J Psychiatr Res. (2022) 150:40–6. doi: 10.1016/j.jpsychires.2022.03.033, PMID: 35349797 PMC8943429

[8] Díez-Cirarda M Yus M Gómez-Ruiz N Polidura C Gil-Martínez L Delgado-Alonso C. Multimodal neuroimaging in post-COVID syndrome and correlation with cognition. Brain. (2023) 146:2142–52. doi: 10.1093/brain/awac384, PMID: 36288544 PMC9620345

[9] García-Sánchez C Calabria M Grunden N Pons C Arroyo JA Gómez-Anson B. Neuropsychological deficits in patients with cognitive complaints after COVID-19. Brain Behav. (2022) 12:3. doi: 10.1002/brb3.2508, PMID: 35137561 PMC8933779

[10] Hadad R Khoury J Stanger C Fisher T Schneer S Ben-Hayun R. Cognitive dysfunction following COVID-19 infection. J Neurovirol. (2022) 28:430–7. doi: 10.1007/s13365-022-01079-y, PMID: 35618983 PMC9134977

[11] Voruz P Allali G Benzakour L Nuber-Champier A Thomasson M Jacot I. Long COVID neuropsychological deficits after severe, moderate, or mild infection. Clin Trans Neurosci. (2022) 6:9. doi: 10.3390/ctn6020009

[12] Bungenberg J Hohenfeld C Costa AS Heine J Schwichtenberg K Hartung T. Characteristic functional connectome related to post-COVID-19 syndrome. Sci Rep. (2024) 14:4997. doi: 10.1038/s41598-024-54554-3, PMID: 38424415 PMC10904373

[13] Chang L Ryan MC Liang H Zhang X Cunningham E Wang J. Changes in brain activation patterns during working memory tasks in people With post-COVID condition and persistent neuropsychiatric symptoms. Neurology. (2023) 100:e2409–23. doi: 10.1212/WNL.0000000000207309, PMID: 37185175 PMC10256123

[14] Douaud G Lee S Alfaro-Almagro F Arthofer C Wang C McCarthy P. SARS-CoV-2 is associated with changes in brain structure in UK biobank. Nature. (2022) 604:697–707. doi: 10.1038/s41586-022-04569-5, PMID: 35255491 PMC9046077

[15] Liang H Ernst T Oishi K Ryan MC Herskovits E Cunningham E. Abnormal brain diffusivity in participants with persistent neuropsychiatric symptoms after COVID-19. Neuroimmune Pharmacol Ther. (2023):37–48. doi: 10.1515/nipt-2022-0016, PMID: 37067870 PMC10091517

[16] Paolini M Palladini M Mazza MG Colombo F Vai B Rovere-Querini P. Brain correlates of subjective cognitive complaints in COVID-19 survivors: a multimodal magnetic resonance imaging study. Eur Neuropsychopharmacol. (2023) 68:1–10. doi: 10.1016/j.euroneuro.2022.12.002, PMID: 36640728 PMC9742225

[17] Scardua-Silva L Amorim da Costa B Karmann Aventurato Í Batista Joao R Machado de Campos B Rabelo de Brito M. Microstructural brain abnormalities, fatigue, and cognitive dysfunction after mild COVID-19. Sci Rep. (2024) 14:1758. doi: 10.1038/s41598-024-52005-7, PMID: 38242927 PMC10798999

[18] Huang S Zhou Z Yang D Wei Zhao M Zeng XX Yanyao D. Persistent white matter changes in recovered COVID-19 patients at the 1-year follow-up. Brain. (2022) 145:1830–8. doi: 10.1093/brain/awab435, PMID: 34918020 PMC8754808

[19] Lu Y Li X Geng D Mei N Pu-Yeh W Huang C-C. Cerebral micro-structural changes in COVID-19 patients–an MRI-based 3-month follow-up study. EClinicalMedicine. (2020) 25:100484. doi: 10.1016/j.eclinm.2020.100484, PMID: 32838240 PMC7396952

[20] Qin Y Jinfeng W Chen T Li J Zhang G Di W. Long-term microstructure and cerebral blood flow changes in patients recovered from COVID-19 without neurological manifestations. J Clin Invest. (2021) 131:e147329. doi: 10.1172/JCI147329, PMID: 33630760 PMC8262559

[21] Huang S Zhou X Zhao W Yanyao D Yang D Huang Y. Dynamic white matter changes in recovered COVID-19 patients: a two-year follow-up study. Theranostics. (2023) 13:724–35. doi: 10.7150/thno.79902, PMID: 36632218 PMC9830428

[22] Esposito F Cirillo M De Micco R Caiazzo G Siciliano M Russo AG. Olfactory loss and brain connectivity after <scp>COVID</Scp> −19. Hum Brain Mapp. (2022) 43:1548–60. doi: 10.1002/hbm.25741, PMID: 35083823 PMC8886650

[23] Dacosta-Aguayo R Puig J Lamonja-Vicente N Carmona-Cervelló M León-Gómez BB Monté-Rubio G. Reduced cortical thickness correlates of cognitive dysfunction in post-COVID-19 condition: insights from a long-term follow-up. Am J Neuroradiol. (2024) 45:647–54. doi: 10.3174/ajnr.A8167, PMID: 38575319 PMC11288549

[24] Goodglass H Kaplan E Barresi B. Test de Boston Para El Diagnóstico de La Afasia. 3rd ed. Madrid: Editorial Médica Panamericana (2001).

[25] Churchill NW Eugenie R Jean Chen J Gilboa A Sekuler A Ji X. Effects of post-acute COVID-19 syndrome on the functional brain networks of non-hospitalized individuals. Front Neurol. (2023) 14:1136408. doi: 10.3389/fneur.2023.1136408, PMID: 37051059 PMC10083436

[26] Greicius MD Krasnow B Reiss AL Menon V. Functional connectivity in the resting brain: a network analysis of the default mode hypothesis. Proc Natl Acad Sci USA. (2003) 100:253–8. doi: 10.1073/pnas.0135058100, PMID: 12506194 PMC140943

[27] Voruz P Cionca A Jacot I de Alcântara A Nuber-Champier GA Benzakour L. Brain functional connectivity alterations associated with neuropsychological performance 6–9 months following <scp>SARS-CoV</Scp> −2 infection. Hum Brain Mapp. (2023) 44:1629–46. doi: 10.1002/hbm.26163, PMID: 36458984 PMC9878070

[28] Zhang H Chung TW-H Wong FK-C Hung IF-N Mak HK-F. Changes in the intranetwork and internetwork connectivity of the default mode network and olfactory network in patients with COVID-19 and olfactory dysfunction. Brain Sci. (2022) 12:511. doi: 10.3390/brainsci12040511, PMID: 35448042 PMC9029634

[29] Cattarinussi G Miola A Trevisan N Valeggia S Tramarin E Mucignat C. Altered brain regional homogeneity is associated with depressive symptoms in COVID-19. J Affect Disord. (2022) 313:36–42. doi: 10.1016/j.jad.2022.06.061, PMID: 35764231 PMC9233546

[30] Dacosta-Aguayo R Lamonja-Vicente N Chacón C Carrasco-Ribelles LA Montero-Alia P Costa-Garrido A. Neurocognitive profile of the post-COVID condition in adults in Catalonia-a mixed method prospective cohort and nested case-control study: study protocol. Vaccine. (2022) 10:849. doi: 10.3390/vaccines10060849, PMID: 35746457 PMC9230542

[31] Soriano JB Murthy S Marshall JC Relan P Diaz JV. A clinical case definition of post-COVID-19 condition by a Delphi consensus. Lancet Infectious Dis. (2021) 22:e102–7. doi: 10.1016/S1473-3099(21)00703-9, PMID: 34951953 PMC8691845

[32] Pena-Casanova J Quinones-Ubeda S Quintana-Aparicio M Aguilar M Badenes D Molinuevo JL. Spanish multicenter normative studies (NEURONORMA project): norms for verbal span, visuospatial span, letter and number sequencing, trail making test, and symbol digit modalities test. Arch Clin Neuropsychol. (2009) 24:321–41. doi: 10.1093/arclin/acp038, PMID: 19661109

[33] Tamayo F Casals-Coll M Sánchez-Benavides G Quintana M Manero RM Rognoni T. Estudios Normativos Españoles En Población Adulta Joven (Proyecto NEURONORMA Jóvenes): Normas Para Las Pruebas Span Verbal, Span Visuoespacial, Letter-Number Sequencing, Trail Making Test y Symbol Digit Modalities Test. Neurologia. (2012) 27:319–29. doi: 10.1016/j.nrl.2011.12.020, PMID: 22405214

[34] Lezak MD Howieson DB Bigler ED Tranel D. Neuropsychological assessment. 5th ed. Oxford University Press. (2012).

[35] Casals-Coll M Sánchez-Benavides G Quintana M Manero RM Rognoni T Calvo L. Estudios Normativos Españoles En Población Adulta Joven (Proyecto NEURONORMA Jóvenes): Normas Para Los Test de Fluencia Verbal. Neurologia. (2013) 28:33–40. doi: 10.1016/j.nrl.2012.02.010, PMID: 22652141

[36] Pena-Casanova J Quinones-Ubeda S Gramunt-Fombuena N Quintana-Aparicio M Aguilar M Badenes D. Spanish multicenter normative studies (NEURONORMA project): norms for verbal fluency tests. Arch Clin Neuropsychol. (2009) 24:395–411. doi: 10.1093/arclin/acp042, PMID: 19648583

[37] Strauss E Sherman E Spreen O. A compendium of neuropsychological tests: administration, norms, and commentary. Neurology. (2006) 41:1856. doi: 10.1212/WNL.41.11.1856-a

[38] Wechsler D. WAIS-III. Madrid: Escala de Inteligencia de Wechsler Para Adultos. Edited by TEA Ediciones (2001).

[39] Schmidt M. Rey auditory verbal learning test: a handbook. Los Angeles, CA: Western Psychological Services (1996).

[40] Palomo R Casals-Coll M Sánchez-Benavides G Quintana M Manero RM Rognoni T. Estudios Normativos Españoles En Población Adulta Joven (Proyecto NEURONORMA Jóvenes): Normas Para Las Pruebas Rey-Osterrieth Complex Figure (Copia y Memoria) y Free and Cued Selective Reminding Test. Neurologia. (2013) 28:226–35. doi: 10.1016/j.nrl.2012.03.008, PMID: 22652140

[41] Pena-Casanova J Gramunt-Fombuena N Quinones-Ubeda S Sanchez-Benavides G Aguilar M Badenes D. Spanish multicenter normative studies (NEURONORMA project): norms for the Rey-Osterrieth complex Figure (copy and memory), and free and cued selective reminding test. Arch Clin Neuropsychol. (2009) 24:371–93. doi: 10.1093/arclin/acp041, PMID: 19661107

[42] Strober LB Bruce JM Arnett PA Alschuler KN DeLuca J Chiaravalloti N. Tired of not knowing what that fatigue score means? Normative data of the modified fatigue impact scale (MFIS). Mult Scler Relat Disord. (2020) 46:102576. doi: 10.1016/j.msard.2020.102576 33296974

[43] Gates TM Cysique LA. The chronicity of HIV infection should drive the research strategy of NeuroHIV treatment studies: a critical review. CNS Drugs. (2016) 30:53–69. doi: 10.1007/s40263-015-0302-7, PMID: 26749584 PMC4733144

[44] Smith SM Jenkinson M Woolrich MW Beckmann CF Behrens TEJ Johansen-Berg H. Advances in functional and structural MR image analysis and implementation as FSL. Neuroimage. (2004) 23:S208–19. doi: 10.1016/j.neuroimage.2004.07.051, PMID: 15501092

[45] Andersson JLR Jenkinson M Smith S. Non-linear optimization. FMRIB Technical Report TR07JA1. (2007). Available at: www.fmrib.ox.ac.uk/analysis/techrep

[46] Pruim RHR Mennes M van Rooij D Llera A Buitelaar JK Beckmann CF. ICA-AROMA: a robust ICA-based strategy for removing motion artifacts from FMRI data. NeuroImage. (2015) 112:267–77. doi: 10.1016/j.neuroimage.2015.02.064, PMID: 25770991

[47] Filippini N MacIntosh BJ Hough MG Goodwin GM Frisoni GB Smith SM. Distinct patterns of brain activity in young carriers of the APOE-Ε4 allele. Proc Natl Acad Sci. (2009) 106:7209–14. doi: 10.1073/pnas.0811879106, PMID: 19357304 PMC2678478

[48] Sharp DJ Beckmann CF Greenwood R Kinnunen KM Bonnelle V De Boissezon X. Default mode network functional and structural connectivity after traumatic brain injury. Brain. (2011) 134:2233–47. doi: 10.1093/brain/awr175 21841202

[49] Nichols TE Holmes AP. Nonparametric permutation tests for functional neuroimaging: a primer with examples. Hum Brain Mapp. (2002) 15:1–25. doi: 10.1002/hbm.1058, PMID: 11747097 PMC6871862

[50] Biswal BB Mennes M Zuo X-N Gohel S Kelly C Smith SM. Toward discovery science of human brain function. Proc Natl Acad Sci. (2010) 107:4734–9. doi: 10.1073/pnas.0911855107, PMID: 20176931 PMC2842060

[51] Smith SM Fox PT Miller KL Glahn DC Mickle Fox P Mackay CE. Correspondence of the Brain’s functional architecture during activation and rest. Proc Natl Acad Sci. (2009) 106:13040–5. doi: 10.1073/pnas.0905267106, PMID: 19620724 PMC2722273

[52] Saltzman LY Longo M Hansel TC. Long-COVID stress symptoms: mental health, anxiety, depression, or posttraumatic stress. Psychol Trauma. (2023). doi: 10.1037/tra0001567, [Online ahead of print] 37561435

[53] Rebsamen M Friedli C Radojewski P Diem L Chan A Wiest R. Multiple sclerosis as a model to investigate <scp>SARS-CoV</Scp> −2 effect on brain atrophy. CNS Neurosci Ther. (2023) 29:538–43. doi: 10.1111/cns.14050, PMID: 36479826 PMC9873510

[54] Hannoun S Kocevar G Durand-Dubief F Stamile C Naji A Cotton F. Evidence of axonal damage in cerebellar peduncles without T2-lesions in multiple sclerosis. Eur J Radiol. (2018) 108:114–9. doi: 10.1016/j.ejrad.2018.09.016, PMID: 30396642

[55] Schoonheim MM Vigeveno RM Rueda FC Lopes PJW Pouwels CH Polman FB. Sex-specific extent and severity of white matter damage in multiple sclerosis: implications for cognitive decline. Hum Brain Mapp. (2014) 35:2348–58. doi: 10.1002/hbm.22332, PMID: 23982918 PMC6869647

[56] Nan Y Knösche TR Zysset S Friederici AD. Cross-cultural music phrase processing: an FMRI study. Hum Brain Mapp. (2008) 29:312–28. doi: 10.1002/hbm.20390, PMID: 17497646 PMC6871102

[57] Raichle ME MacLeod AM Snyder AZ Powers WJ Gusnard DA Shulman GL. A default mode of brain function. Proc Natl Acad Sci USA. (2001) 98:676–82. doi: 10.1073/pnas.98.2.676, PMID: 11209064 PMC14647

[58] Yi L Wang J Jia L Zhao Z Jie L Li K. Structural and functional changes in subcortical vascular mild cognitive impairment: a combined voxel-based morphometry and resting-state FMRI study. PLoS One. (2012) 7:e44758. doi: 10.1371/journal.pone.0044758, PMID: 23028606 PMC3446994

[59] Ikonomidis I Lambadiari V Mitrakou A Kountouri A Katogiannis K Thymis J. Myocardial work and vascular dysfunction are partially improved at 12 months after <scp>COVID</Scp> −19 infection. Eur J Heart Fail. (2022) 24:727–9. doi: 10.1002/ejhf.2451, PMID: 35138689 PMC9087421

[60] Kirschenbaum D Imbach LL Rushing EJ Frauenknecht KBM Gascho D Ineichen BV. Intracerebral Endotheliitis and microbleeds are neuropathological features of COVID-19. Neuropathol Appl Neurobiol. (2021) 47:454–9. doi: 10.1111/nan.12677, PMID: 33249605 PMC7753688

[61] Meyer PT Hellwig S Blazhenets G Hosp JA. Molecular imaging findings on acute and long-term effects of COVID-19 on the brain: a systematic review. J Nucl Med. (2022) 63:971–80. doi: 10.2967/jnumed.121.263085, PMID: 35177424 PMC9258567

[62] Reichard RR Kashani KB Boire NA Constantopoulos E Guo Y Lucchinetti CF. Neuropathology of COVID-19: a Spectrum of vascular and acute disseminated encephalomyelitis (ADEM)-like pathology. Acta Neuropathol. (2020) 140:1–6. doi: 10.1007/s00401-020-02166-2, PMID: 32449057 PMC7245994

[63] Siepmann T Sedghi A Simon E Winzer S Barlinn J de With K. Increased risk of acute stroke among patients with severe COVID-19: a multicenter study and Meta-analysis. Eur J Neurol. (2021) 28:238–47. doi: 10.1111/ene.14535, PMID: 32920964

[64] Blazhenets G Schroeter N Bormann T Thurow J Wagner D Frings L. Slow but evident recovery from neocortical dysfunction and cognitive impairment in a series of chronic COVID-19 patients. J Nucl Med. (2021) 62:910–5. doi: 10.2967/jnumed.121.262128, PMID: 33789937 PMC8882885

[65] World Health Organization. (2010). A healthy lifestyle - who recommendations. Available at: https://www.who.int/europe/news-room/fact-sheets/item/a-healthy-lifestyle---who-recommendations (Accessed June 24, 2023).

