# Supplementary material for: Multimodal neuroimaging in Long-COVID and its correlates with cognition 1.8 years after SARS-CoV-2 infection: a cross-sectional study of the Aliança ProHEpiC-19 Cognitiu
Source: Front Neurol. 2024 Sep 13;15:1426881. doi: 10.3389/fneur.2024.1426881 (PMC11428557; doi:10.3389/fneur.2024.1426881)
Supplement: Supplementary file 1 [file Data_Sheet_1.docx]

Supplementary Material

# Supplementary Data

**Study procedure.**

**Sociodemographic and neuropsychological assessment information**

**Sociodemographic**

Sex (Male, Female), age (years), education level (Elementary, Secondary, High School, University Degree, Specialist or Master, Doctorate), job field (Doctor, Nurse, Health Services, Health Assistants, Other), weight (kg), height (cm), body mass index (according to the WHO standards (“World Health Organization, ‘A Healthy Lifestyle - WHO Recommendations,’ 06-May-2010. [Online]. Available: Https://Www.Who.Int/Europe/News-Room/Fact-Sheets/Item/a-Healthy-Lifestyle---Who-Recommendations. [Accessed: 24-Jun-2023].,” n.d.): Underweight, Normal Weight, Overweight, Obesity Class I, Obesity Class II, Obesity Class III) high blood pressure (Yes, No), cholesterol (Yes, No), diabetes (Yes, No), tobacco (Never, Smoker, Ex-smoker), and alcohol consumption (Yes, No). During this first session, all participants completed a comprehensive neuropsychological assessment (see below). They also reported their COVID-19 experience (date and method of diagnosis, clinical spectrum that includes symptoms and treatment). In the second session, PCC individuals underwent a brain MRI scan acquisition within six months from the cognitive assessment. To protect the privacy of our participants, we used cryptographic hashtags to anonymize the project database. We also employed the same 10-digit numeric encoding system hosted by REDCap, version 12.4.22 - Vanderbilt University, for the MRI study.

**Acquisition Sequences and Parameters**

The MRI protocol included a 3D magnetization-prepared rapid gradient echo (MP-RAGE) T1-weighted protocol (repetition time [TR]: 2000 ms; inversion time [TI]: 1500 ms; echo time [TE]: 2.7 ms; flip angle: 15°) reconstructed in the sagittal plane (158 slices; field of view: 256x256 mm; and voxel size: 1×1×1 mm3) and a Fluid Attenuated Inversion Recovery (FLAIR) image (TR: 5000 ms; TE: 504 ms; TI: 1500 ms; flip angle: 15°), acquired in the sagittal plane (72 slices with no gap; field of view: 256x256 mm; and voxel size: 1×1×2 mm3). DWI was acquired in 48 non-collinear diffusion directions, with a b-value of 1.000 s/mm2, with the following an echo planar imaging (EPI) protocol (TR: 3600 ms; TE: 81 ms; flip angle: 90º), acquired in the axial plane (field of view: 240×256 mm; and voxel size: 2×2×2 mm3; phase-encoding direction: PA). Two B0 volumes with a b-value of 0 s/mm2 and opposite phase-encoding directions (AP and PA) were also acquired. Resting-state blood oxygen level-dependent (BOLD) functional MRI was acquired using an EPI protocol (TR: 2.25 s; TE: 25 ms; flip angle: 90º; acquisition time: 9:49 min; 256 volumes), acquired in axial plan plane (42 slices with no gap; field of view: 240×240 mm; and voxel size: 3×3×3 mm3). Participants were instructed to remain still with their eyes closed but stay awake.

| Coefficients^a^ | | | | | | | | |
| --- | --- | --- | --- | --- | --- | --- | --- | --- |
| Model: **Memory domain** | | Unstandardized Coefficients | | Standardized Coefficients | t | Sig. | 95,0% Confidence Interval for B | |
|  |  | B | Std. Error | Beta |  |  | Lower Bound | Upper Bound |
| 1 | (Constant) | 0,205 | 1,091 |  | 0,187 | 0,852 | -1,996 | 2,406 |
|  | sex_n | -0,888 | 0,396 | -0,354 | -2,245 | **0,030** | -1,686 | -0,090 |
|  | age | -0,025 | 0,013 | -0,282 | -1,889 | **0,066** | -0,051 | 0,002 |
|  | Educ | 0,089 | 0,044 | 0,288 | 2,028 | **0,049** | 0,001 | 0,178 |
|  | BMI | 0,002 | 0,018 | 0,016 | 0,114 | 0,910 | -0,035 | 0,039 |
|  | hta | 0,100 | 0,306 | 0,047 | 0,327 | 0,745 | -0,517 | 0,717 |
|  | col | -0,068 | 0,278 | -0,035 | -0,246 | 0,807 | -0,629 | 0,493 |
|  | diab | 0,013 | 0,886 | 0,002 | 0,015 | 0,988 | -1,773 | 1,799 |
|  | smok | -0,318 | 0,418 | -0,105 | -0,759 | 0,452 | -1,161 | 0,526 |
|  | alch | 0,328 | 0,244 | 0,202 | 1,345 | 0,186 | -0,164 | 0,820 |
| a. Dependent Variable: Memory; hta = hypertension; diab = diabetes; smok = smoking; alch = alcohol consumption; col = cholesterolemia; BMI = Body Mass Index. | | | | | | | | |

| Coefficients^a^ | | | | | | | | |
| --- | --- | --- | --- | --- | --- | --- | --- | --- |
| Model: **Visuospatial and Visuoconstructive** | | Unstandardized Coefficients | | Standardized Coefficients | t | Sig. | 95,0% Confidence Interval for B | |
|  |  | B | Std. Error | Beta |  |  | Lower Bound | Upper Bound |
| 1 | (Constant) | 2,251 | 1,094 |  | 2,057 | 0,046 | 0,044 | 4,457 |
|  | sex_n | -0,807 | 0,397 | -0,327 | -2,034 | **0,048** | -1,607 | -0,007 |
|  | age | -0,019 | 0,013 | -0,224 | -1,467 | 0,150 | -0,046 | 0,007 |
|  | Educ | 0,014 | 0,044 | 0,047 | 0,323 | 0,748 | -0,075 | 0,103 |
|  | BMI | -0,030 | 0,018 | -0,234 | -1,611 | 0,115 | -0,067 | 0,007 |
|  | hta | 0,345 | 0,307 | 0,166 | 1,124 | 0,267 | -0,274 | 0,964 |
|  | col | -0,106 | 0,279 | -0,055 | -0,381 | 0,705 | -0,669 | 0,456 |
|  | diab | 0,110 | 0,888 | 0,019 | 0,124 | 0,902 | -1,681 | 1,901 |
|  | smok | -0,228 | 0,419 | -0,077 | -0,543 | 0,590 | -1,074 | 0,618 |
|  | alch | 0,042 | 0,245 | 0,026 | 0,172 | 0,864 | -0,451 | 0,535 |
| a. Dependent Variable: Visuospatial and Visuoconstructive; hta = hypertension; diab = diabetes; smok = smoking; alch = alcohol consumption; col = cholesterolemia; BMI = Body Mass Index. | | | | | | | | |

| Coefficients^a^ | | | | | | | | |
| --- | --- | --- | --- | --- | --- | --- | --- | --- |
| Model: **Attention** | | Unstandardized Coefficients | | Standardized Coefficients | t | Sig. | 95,0% Confidence Interval for B | |
|  |  | B | Std. Error | Beta |  |  | Lower Bound | Upper Bound |
| 1 | (Constant) | -2,076 | 0,945 |  | -2,197 | 0,033 | -3,983 | -0,170 |
|  | sex_n | 0,660 | 0,343 | 0,328 | 1,925 | **0,061** | -0,031 | 1,351 |
|  | age | 0,012 | 0,011 | 0,165 | 1,020 | 0,313 | -0,011 | 0,034 |
|  | Educ | 0,039 | 0,038 | 0,158 | 1,032 | 0,308 | -0,038 | 0,116 |
|  | BMI | -0,010 | 0,016 | -0,097 | -0,630 | 0,532 | -0,042 | 0,022 |
|  | hta | 0,117 | 0,265 | 0,069 | 0,443 | 0,660 | -0,417 | 0,652 |
|  | col | -0,009 | 0,241 | -0,006 | -0,038 | 0,970 | -0,495 | 0,477 |
|  | diab | -0,412 | 0,767 | -0,088 | -0,537 | 0,594 | -1,959 | 1,135 |
|  | smok | 0,037 | 0,362 | 0,016 | 0,103 | 0,918 | -0,693 | 0,768 |
|  | alch | -0,043 | 0,211 | -0,033 | -0,202 | 0,841 | -0,469 | 0,384 |
| a. Dependent Variable: Attention; hta = hypertension; diab = diabetes; smok = smoking; alch = alcohol consumption; col = cholesterolemia; BMI = Body Mass Index. | | | | | | | | |

| Coefficients^a^ | | | | | | | | |
| --- | --- | --- | --- | --- | --- | --- | --- | --- |
| Model: **Executive Functions** | | Unstandardized Coefficients | | Standardized Coefficients | t | Sig. | 95,0% Confidence Interval for B | |
|  |  | B | Std. Error | Beta |  |  | Lower Bound | Upper Bound |
| 1 | (Constant) | -1,166 | 0,677 |  | -1,722 | 0,092 | -2,532 | 0,200 |
|  | sex_n | 0,256 | 0,246 | 0,180 | 1,043 | 0,303 | -0,239 | 0,751 |
|  | age | -0,001 | 0,008 | -0,011 | -0,069 | 0,945 | -0,017 | 0,016 |
|  | Educ | 0,028 | 0,027 | 0,161 | 1,036 | 0,306 | -0,027 | 0,084 |
|  | BMI | 0,002 | 0,011 | 0,021 | 0,136 | 0,893 | -0,021 | 0,024 |
|  | hta | -0,329 | 0,190 | -0,275 | -1,734 | 0,090 | -0,712 | 0,054 |
|  | col | -0,023 | 0,173 | -0,021 | -0,132 | 0,896 | -0,371 | 0,325 |
|  | diab | 0,413 | 0,550 | 0,125 | 0,751 | 0,457 | -0,695 | 1,521 |
|  | smok | 0,085 | 0,260 | 0,050 | 0,326 | 0,746 | -0,439 | 0,608 |
|  | alch | 0,019 | 0,151 | 0,021 | 0,126 | 0,900 | -0,286 | 0,324 |
| a. Dependent Variable: Executive Functions; hta = hypertension; diab = diabetes; smok = smoking; alch = alcohol consumption; col = cholesterolemia; BMI = Body Mass Index. | | | | | | | | |

| Coefficients^a^ | | | | | | | | |
| --- | --- | --- | --- | --- | --- | --- | --- | --- |
| Model: **Language** | | Unstandardized Coefficients | | Standardized Coefficients | t | Sig. | 95,0% Confidence Interval for B | |
|  |  | B | Std. Error | Beta |  |  | Lower Bound | Upper Bound |
| 1 | (Constant) | -1,068 | 0,909 |  | -1,176 | 0,246 | -2,901 | 0,764 |
|  | sex_n | -0,283 | 0,330 | -0,142 | -0,858 | 0,396 | -0,947 | 0,382 |
|  | age | 0,008 | 0,011 | 0,116 | 0,741 | 0,462 | -0,014 | 0,030 |
|  | Educ | 0,103 | 0,037 | 0,418 | 2,802 | **0,008** | 0,029 | 0,177 |
|  | BMI | 0,015 | 0,015 | 0,146 | 0,972 | 0,337 | -0,016 | 0,046 |
|  | hta | -0,098 | 0,255 | -0,058 | -0,384 | 0,703 | -0,612 | 0,416 |
|  | col | -0,270 | 0,232 | -0,174 | -1,167 | 0,250 | -0,738 | 0,197 |
|  | diab | -0,048 | 0,738 | -0,010 | -0,065 | 0,948 | -1,536 | 1,439 |
|  | smok | 0,172 | 0,348 | 0,072 | 0,493 | 0,625 | -0,531 | 0,874 |
|  | alch | -0,068 | 0,203 | -0,053 | -0,333 | 0,740 | -0,477 | 0,342 |
| a. Dependent Variable: Language; hta = hypertension; diab = diabetes; smok = smoking; alch = alcohol consumption; col = cholesterolemia; BMI = Body Mass Index. | | | | | | | | |

**
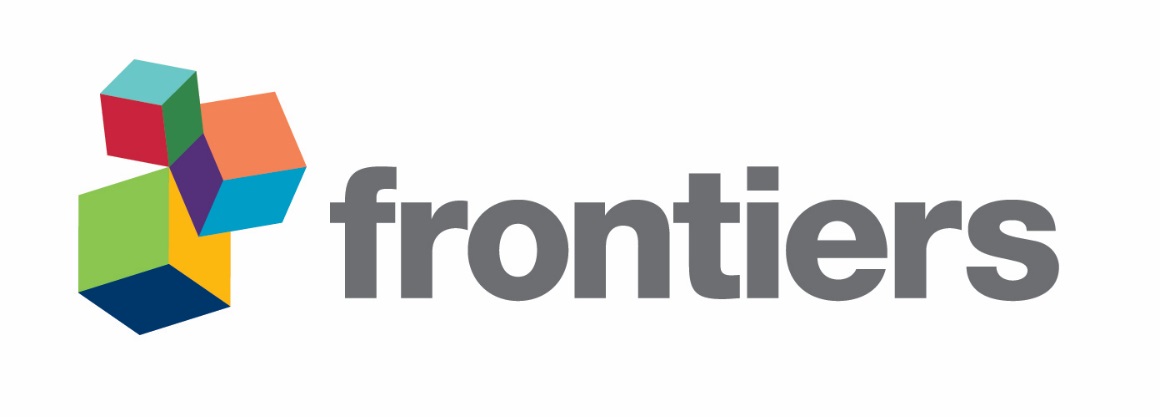
**
